# Enhanced Raman Scattering from Vibro-Polariton Hybrid States**

**DOI:** 10.1002/anie.201502979

**Published:** 2015-06-03

**Authors:** Atef Shalabney, Jino George, Hidefumi Hiura, James A Hutchison, Cyriaque Genet, Petra Hellwig, Thomas W Ebbesen

**Affiliations:** ISIS & icFRC, University of Strasbourg and CNRS8 allée Gaspard Monge, 67000 Strasbourg (France); Smart Energy Research Laboratories, NEC CorporationTsukuba (Japan); CMC, University of Strasbourg and CNRS1 rue Blaise Pascal, 67070 Strasbourg (France)

**Keywords:** optical cavity, Raman scattering, strong coupling, vibrations, vibro-polariton states

## Abstract

Ground-state molecular vibrations can be hybridized through strong coupling with the vacuum field of a cavity optical mode in the infrared region, leading to the formation of two new coherent vibro-polariton states. The spontaneous Raman scattering from such hybridized light–matter states was studied, showing that the collective Rabi splitting occurs at the level of a single selected bond. Moreover, the coherent nature of the vibro-polariton states boosts the Raman scattering cross-section by two to three orders of magnitude, revealing a new enhancement mechanism as a result of vibrational strong coupling. This observation has fundamental consequences for the understanding of light-molecule strong coupling and for molecular science.

Raman spectroscopy uses excitations in the visible region to acquire vibrational fingerprints of molecules, making it a powerful tool for chemical analysis and molecular detection. A major breakthrough was the discovery of the surface-enhanced Raman scattering (SERS) effect[1–3] in the range of 10^10^–10^14^, caused by extremely high electromagnetic fields associated with localized plasmonic resonances, which enable even single-molecule detection.[4], [5] All these attributes have made Raman scattering a widely used tool for a broad range of disciplines, including chemistry, physics, and life sciences.[6–8]

Hybrid light–matter states are formed when a molecular transition and a resonant optical mode enter the so-called strong-coupling regime, whereby they exchange energy faster than any relaxation process.[9–30] Two new hybrid states are generated, both having features of light and matter, called polaritonic states. They are separated by the Rabi splitting *ħω_VR_*, as illustrated in Figure 1, which is proportional to $[\sqrt N =$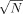, with *N* being the number of molecules coupled to the optical mode. The strong coupling process involves the zero-point fluctuations of the cavity and the molecular transition; therefore it occurs even in the dark. The light–matter hybridization is expected to alter the properties of the system. Indeed it has been shown that by strongly coupling molecular electronic transitions with a cavity or plasmonic mode, properties such as a photochemical isomerization reaction rate,[24] the conductivity[21] and work function[25] of organic materials can be modified. However, light–matter strong coupling is not limited to electronic transitions, which has been extensively studied during the last two decades.

**Figure 1:**
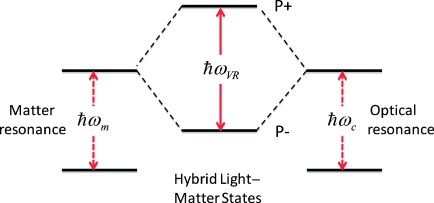
Schematic illustration of strong coupling between a molecular resonance with frequency *ω_m_* and a cavity mode with frequency *ω_c_*, generating new light–matter hybrid states P+ and P−, separated by the vacuum Rabi splitting *ħω_VR_*.

Recently, we were able to show for the first time that vibrational transitions in the ground state can also be strongly coupled to an optical mode using microcavities in the infrared (IR) region.[28], [29] The resonant coupling is achieved by tuning the frequency *ω_c_* of microcavities to a given molecular vibrational frequency *ω_v_* of a specific bond in the molecule, resulting in the formation of two new hybrid vibro-polariton states VP+ and VP− with energies *ħω*_+_ and *ħω*_−_, respectively (as detailed in Figure 2 a).

**Figure 2:**
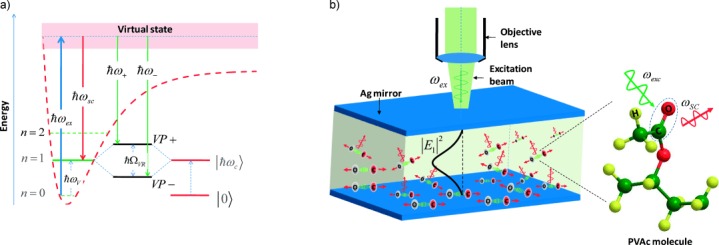
a) Energy-level diagram under vibrational strong coupling showing the scheme for the Raman scattering from the new vibro-polariton states. b) Schematic description of the FP cavity used in this study composed of two thin Ag mirrors spaced by a PVAc layer. Raman excitation performed using a micro-Raman system with an objective lens. The inset shows the 3D structure of the PVAc monomer emphasizing the C=O bond, the target for VSC, with the exciting and Stokes scattered photons generating Raman scattering from the coupled vibration. |*E*_1_|^2^ (black line) shows qualitatively the intensity distribution of the first cavity mode with which the C=O resonators are coupled.

One of the fundamental debates in molecular strong coupling is whether the observed collective Rabi splitting generated by coupling simultaneously to *N* molecules occurs at the level of each molecule. It was sometimes argued that each molecule experiences a much smaller effect on its local energy landscape corresponding to the observed collective Rabi splitting redivided by $[\sqrt N =$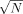. If this were the case, the effect of strong coupling on each molecule would be negligible and could not explain the significant changes in the observed properties. The better analogy of the collective polaritonic states is that of molecular orbitals that are delocalized across all the contributing atoms with immediate consequences for chemistry.

Here, we provide insight into this issue by measuring for the first time the spontaneous Raman scattering from molecules under vibrational strong coupling (VSC), as it probes the property at the level of each molecule from a spectral window outside that involved in the strong coupling. We found that the Rabi splitting at the level of single molecule is indeed on the scale of the collective coupling strength. Surprisingly, there is 10^2^ to 10^3^ enhancement in the scattering cross-sections for the coupled molecules relative to the uncoupled ones.

To explore the Raman properties of molecules under vibrational strong coupling, we needed to choose a system with a vibrational transition that is active in both IR and Raman spectroscopy. The IR absorption was necessary to achieve strong coupling and the system could then be studied nonresonantly by Raman scattering (see Figure 2). Polyvinyl acetate (PVAc) has such a feature where the C=O bond has a symmetric stretching frequency at 1740 cm^−1^ (215 meV; red curve in Figure 3 a), which can be strongly coupled, as already demonstrated.[28] In order to achieve VSC with the C=O vibration transition, a Fabry–Perot (FP) cavity was prepared by forming a film of the polymer between two thin silver mirrors (see details on sample preparation in the Supporting Information and Figure 2 b). The thickness of the PVAc layer determined that of the cavity and therefore the frequency of the cavity mode so that the system could be studied in and out of the VSC regime. Raman scattering signals were measured in reflection, using a micro-Raman system with excitation wavelength of 514 nm and numerical aperture (NA) of about 0.7 in both excitation and collection. This excitation wavelength is thus very far from the VSC condition at 1740 cm^−1^ (5.7 μm).

**Figure 3:**
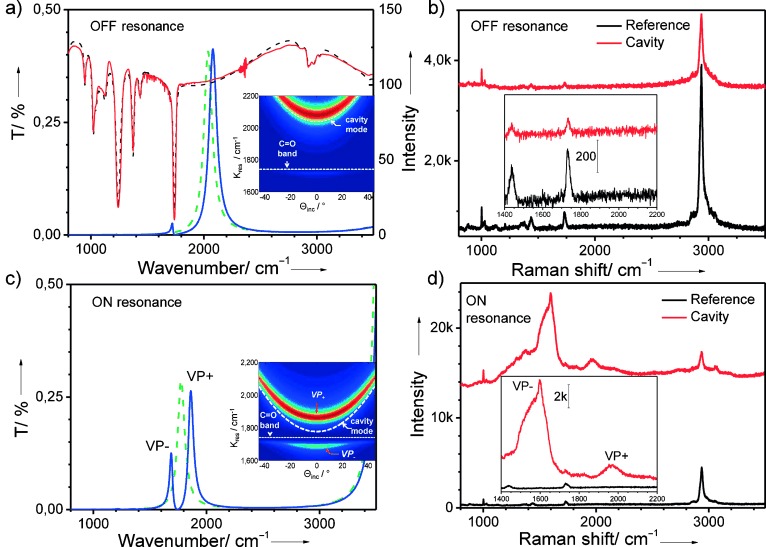
a) Red and dashed black curves are the measured and calculated transmission of a thin PVAc film (about 2 µm) deposited on Ge substrate. The green dashed line shows the fundamental cavity mode after deactivating all the absorption bands of the PVAc, leaving only the background refractive index of the cavity. The blue curve is the transmission of the coupled cavity. The inset shows the C=O band and the uncoupled cavity mode without the C=O resonators (white dashed curve) superimposed on the dispersion of the cavity after adding the C=O resonators. b) Raman scattering from the cavity that is not in the VSC regime given in (a) (red spectrum) and the reference sample (black spectrum). c) Cavity transmission without C=O resonators (dashed green) and with C=O resonators (solid blue) showing the formation of VP+ and VP− in the VSC regime. The inset shows in color plot the dispersion of VP+ and VP− with the dispersion of the uncoupled C=O resonators (dashed white line) and the uncoupled cavity mode (dashed parabola). d) Raman scattering from the cavity given in (c) showing in the inset the new density of states at 1966 cm^−1^ and 1600 cm^−1^ which correspond to the generation of VP+ and VP−, respectively. The Raman signals are vertically shifted for clarity.

Figure 3 also shows the IR transmission spectra and Stokes Raman scattering signals from two cavities, which are off- and on-resonance with the C=O mode. In the case of the off-resonance cavity (Figure 3 a and b), the dashed green and solid blue curves describe the transmission of the cavity in the absence and presence of the C=O resonators, respectively, showing that there is no VSC signature. The first fundamental mode of the cavity at 2036 cm^−1^ in the absence of the C=O resonators shifts slightly to higher frequency because of local modification in the refractive index as a result of the C=O oscillators inside the cavity. In this off-resonance case, the dispersion given in the inset of Figure 3 a does not show any formation of new branches, as expected. Raman scattering from the off-resonance cavity and its reference (the same structure as the cavity without the top mirror) are presented in Figure 3 b. One can see that except for a decrease in the intensities of the bands, there are no detectable new features in the cavity with respect to the reference.

When the cavity thickness is tuned to couple to the C=O vibrational frequency (as shown in Figure 3 c), clear splitting in the transmission spectrum demonstrates the VSC regime. The cavity fundamental mode in the coupled system comes at around 1770 cm^−1^ (as shown by the dashed green curve in Figure 3 c), which leads to the formation of two peaks at 1842 cm^−1^ and 1681 cm^−1^ for VP+ and VP−, respectively. The inset in Figure 3 c confirms the Rabi splitting and the dispersion of the hybridized states. Simultaneously, new features in the Raman signal are clearly observed in Figure 3 d, appearing as a new density of states peaked at 1966 cm^−1^ and 1600 cm^−1^. These new peaks are not present in the Raman scattering of the reference and can only originate from the formation of VP+ and VP− in the coupled system. The large width of the VP+ and VP− Raman peaks are due to the combination of the high NA used in the micro-Raman apparatus with the dispersive nature of the hybrid states (inset Figure 3 c). In the Raman signal from the on-resonance cavity, there is still a detectable signal at 1730 cm^−1^ which comes from the uncoupled molecules. This reservoir of uncoupled molecules exists because of the random distribution of orientations of the targeted bond, which implies that not all the molecules are strongly coupled.[27] In addition, the field strength is not uniform inside the cavity because of the mode structure, so some molecules can find themselves at locations where the field is so weak that VSC will not occur (see Figure 2 b). The asymmetry in VP− stems from the presence of other vibrational modes that overlap with the new density of state. The very weak band at 1595 cm^−1^, for instance, appears as a sharp tip on VP−, whereas those at 1436 cm^−1^ and 1380 cm^−1^ broaden the left shoulder of VP−.

The possibility of having photo damage and carbonization in the samples was ruled out by examining the stability of the bare molecule layer under different excitation conditions (see the Supporting Information for details) and the fact that the other bands remain unchanged.

The Raman data under VSC in Figure 3 d shows two remarkable features. First, the intensities of the VP+ and VP− Raman scattering peaks are two to three orders of magnitude larger than the uncoupled C=O band intensity. Second, the Rabi splitting is approximately two times larger than the IR data taken on the same sample. Before discussing these features, we will analyze the effect of fine-tuning the cavity around the vibrational C=O peak on the Raman scattering (the method is described in the Supporting Information).

Figure 4 a shows that the IR spectra of different cavities have their fundamental mode (the dashed curves) at slightly different frequencies relative to the C=O absorption band (dotted black curve). The solid curves show the corresponding VP bands in each of the cavities. In Figure 4 b, one can clearly see that the Raman scattering intensity from the new peaks strongly depends on the fine-tuning of the cavity mode with respect to the absorption band. This observation confirms again that the new peaks only result from the VP states

**Figure 4:**
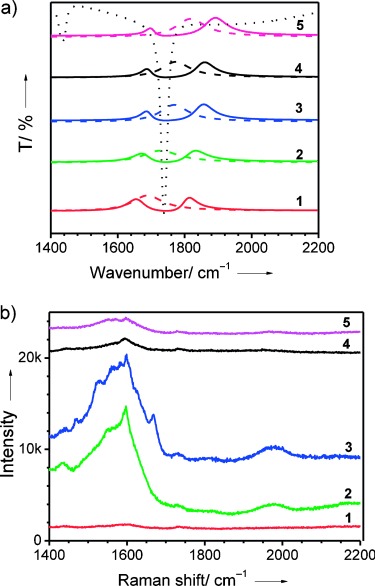
a) Transmission spectra of different cavities tuned around the C=O absorption band (black dotted curve). The detuning from the absorption can be seen by slightly shifting the uncoupled cavity modes (dashed colored curves) with respect to the C=O band at 1740 cm^−1^. b) Raman scattering from the same cavities with corresponding numbers as in (a), showing the change in Raman intensity from VP+ and VP− versus fine-tuning the cavity mode around the C=O absorption band. The spectra in (a) and (b) are shifted for clarity.

In order to quantitatively analyze the new states and considering the experimental set-up, the intensity of the scattered Stokes photons (*I*_S_)_*i*_ collected for vibration *i* with frequency *ω*_S_(*ω_i_*=*ω*_ex_−*ω*_S_) can be described by:

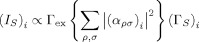
(1)

where *Γ*_ex_ is the optical excitation distribution function related to the mode structure at the excitation wavelength *ω*_ex_, (*α*_*ρσ*_)_*i*_ the matrix element of the polarizability tensor for vibrational transition *i* where *ρ*,*σ*=*x*,*y*,*z* independently refer to the molecule fixed coordinate system; (*Γ*_S_)_*i*_ is introduced to account for the transmission through the system of the Stokes photon from the vibrating molecule to the detector. The main point of Equation (1) is that both *Γ*_ex_ and (*Γ*_S_)_*i*_ are optical structure-dependent quantities that can be evaluated given the geometrical parameters of the cavity, while (*α*_*ρσ*_)_*i*_ is an intrinsic property of the vibrating bond, which solely depends on the properties of the specific scattering state.

It is important to note that the cavities are tuned to have their first mode around 1740 cm^−1^ (corresponding to a wavelength of 5.7 μm), while both the excitation and the Stokes wavelengths are in the visible region and therefore fall in the spectral region of higher modes of the cavity. Furthermore, the confinement of the cavities in the visible region is very low as a result of the thin mirrors and the extremely low *Q* factors of the cavities considered in this study. All the above observations imply that both *Γ*_ex_ and (*Γ*_S_)_*i*_ are close to unity (as detailed in the Supporting Information). This can be further confirmed by observing other peaks in Raman signals that are not involved in the VSC, which do not change much in the cavity compared to the reference. This means that, according to Equation (1), the amplification factor *G*_*αi*_ observed under VSC in the Raman scattering intensities of VP+ and VP− relative to the uncoupled vibration is simply given by the ratio of the polarizability tensor elements in these two conditions:

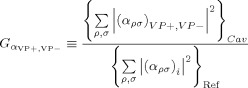
(2)

where the subscripts VP+, VP− represent the hybrid states formed by strongly coupled vibration *i*. Careful estimation for the enhancement factors of VP+ and VP− give polarizability amplification factors *G*_*α*_ of two and three orders of magnitude, respectively. Although this result needs to be addressed by a rigorous theoretical analysis of such a strongly coupled system, in the following we speculate on the origin of this surprising finding.

Under light–matter strong coupling, the spatial coherence imposed by the single-mode cavity field drives all the coupled resonators in phase with each other, as demonstrated experimentally for exciton-polaritons.[16], [17] With this consideration in mind, one possibility is that the Raman signal under VSC is boosted by constructive interference. Alternatively, it is the polarizability per molecule that is enhanced by the extended coherence of the polaritonic states. This is in analogy with the enhanced polarizability observed in molecular clusters as a result of dipole–dipole interaction.[31]

The simplest explanation for the difference in amplification factors for VP+ and VP− can be found in the combination of the high NA necessary in micro-Raman spectroscopy and the dispersive nature of the VPs (see inset in Figure 3 c). Under those experimental conditions, Raman spectroscopy interrogates the sample over a large range of k vectors with different consequences for VP+ and VP−. At resonance, the vibrational and photonic content of VP+ and VP− are identical. For higher values of k, the VP+ nature becomes more photonic, while VP− becomes more material (i.e. vibrational; see the Supporting Information for details). As the Raman signal originates from scattering on the vibrational component, the integration over a range of k values will result in higher scattering from VP− than from VP+.

These results are very different from those of earlier studies of spontaneous and stimulated[32], [33] Raman scattering in optical resonators, whether it involved polaritons or not. In all those cases, the aim was to enhance the Raman scattering of a given vibration by enhancing the electromagnetic field, that is, the first and third terms of Equation (1) were boosted. Even in the context of cavity exciton-polaritons, the cavity polariton modes were exploited to enhance Raman signals by tuning the excitation frequency, Stokes line, or both to be in resonance with the mixed exciton-cavity-mode states.[34–28]

In contrast, here it is the Raman scattering cross-section that is amplified under VSC. This provides a new approach to boosting Raman scattering, which could possibly be combined with field enhancement for practical applications, such as enhancing specific vibrations in SERS and TERS selectively by strongly coupling them to the plasmon or gap modes. Another highly unusual feature of our results is the fact that the Rabi splitting between the new states observed in Raman scattering is twice that recorded in the IR spectra, looking much like an overtone. While the latter represents the collective response of the entire system without distinguishing between the different species of the sample, Raman spectroscopy gives a signature of local and individual resonators. It raises the question of whether the selection rules are the same for VSC in Raman and in IR spectroscopy, which will require theoretical studies beyond the scope of this report.

The direct observation of a large Rabi splitting in the Raman scattering confirms that light–matter hybridization can significantly modify the vibrational frequency of chemical bonds. Together with the demonstration that VSC can also be done in the liquid phase in microfluidic cavities[29] makes it worthwhile to check whether the rate of a chemical reaction can be modified in the ground state under VSC. This could potentially open a whole new approach to controlling chemical reactions by selectively coupling vibrations of specific bonds and be useful as a tool to understand reaction mechanisms.
