# Supplementary material for: Enhanced Raman Scattering from Vibro-Polariton Hybrid States
Source: Angew Chem Int Ed Engl. 2015 Jun 3;54(27):7971–5. doi: 10.1002/anie.201502979 (PMC4515085; doi:10.1002/anie.201502979)
Supplement: Supplementary file 1 [file anie0054-7971-sd1.pdf]

Angewandte Chemie
Eine Zeitschrift der Gesellschaft Deutscher Chemiker

Supporting Information

Enhanced Raman Scattering from Vibro-Polariton Hybrid States**

*Atef Shalabney, Jino George, Hidefumi Hiura, James A. Hutchison, Cyriaque Genet, Petra Hellwig, and Thomas W. Ebbesen**

anie_201502979_sm_miscellaneous_information.pdf

Table of contents:

1. Samples preparation for IR and Raman measurements

2. Samples characterization in the visible region

3. Assessment of the enhancement factors for the vibro-polariton states

4. Composition of vibro-polariton states

5. References

1. Samples preparation for IR and Raman measurements

All the cavities in this work were prepared using Ag thin mirrors and Poly Vinyl Acetate (PVAc) as the cavity medium when the targeted vibrational mode for strong coupling (SC) is the C=O symmetric stretching band.

An approximately 10 nm- thick Ag mirror was sputtered on a clean Ge substrate. Then, a PVAc film was deposited by spin-casting to form a layer of about 2 microns thickness tuned to overlap the first cavity mode with the (C=O) bond stretching band of PVAc at 1740 cm^{-1}. The cavity mode was tuned to be in or out of strong coupling (SC) with the C=O band by varying the polymer film thickness. Finally, the cavity was formed by sputtering a second Ag layer (10 nm) directly on top of the polymer.

PVAc (MW: 140000) was dissolved in Toluene (15 wt. %) and mechanically steered at 100 °C for 40 hours, cooled to room temperature and passed thru a 0.22 μm Nylon filter prior to spin-casting. The thickness of the Ag mirrors was optimized to obtain sufficient intensity in the transmission spectra in the required spectral region. Due to the significant increase of both the real and imaginary parts of the dielectric constant of Ag in the infra-red (IR) region, a compromise was necessary between the cavity quality factor and the transmission mode intensity.

To measure the net polymer absorption without the cavity, another sample of the same thickness was prepared by spin-casting the PVAc solution directly on top of a clean Ge substrate.

The spectra of the cavity were acquired by standard FTIR (Fourier transform infra-red) spectrometer (Nicolet 6700) in transmission mode. Prior to every measurement, a background

was acquired in order to normalize the actual measurement and avoid baseline instability. All the measurements were performed with a resolution of 1 cm^{-1} and averaged over 128 scans to enhance the signal to noise (SNR) in the spectral range 400-7400 cm^{-1}.

The dispersion of the two hybrid states of the coupled system was measured by varying the incidence angle in the range from -60° to +60°. The position of the bare cavity mode was tuned by varying the in-plane wave vector k_x with the incidence angle ($k = 10^4/(2n_c L\cos\theta_i)$) whereas k is the first cavity mode wave-vector in cm^{-1}, n_c the background polymer refractive index, L the cavity thickness, and θ_i the incidence angle.

By measuring the IR spectrum, the thickness of the cavity was macroscopically determined and the cavity first mode represent the average response of the entire probed area which is about 5 mm in diameter.

1.1 Fabricating cavities for the Raman measurements

After calibrating the required cavity thickness to be either on-resonance or off-resonance with the C=O absorption band, the cavity was fabricated again with the same thickness but on glass substrates which are easier to handle for the Raman studies and it was verified that it gives the same results. Raman scattering was measured in reflection mode using “inVia” confocal Raman microscope (Renishaw) equipped with 50x objective lens and a laser power of 25mW at the 514 nm excitation wavelength. The spot size on the sample was measured and found to be around 80 μm with the full laser power.

The stability of the samples was confirmed by examining the Raman signal from the bare molecule layer under different excitation intensities and number of scans. The spectra in Fig. S1 show that no features of photo-damage or carbonization of the PVAc layer is present. This would typically be characterized by the evolution of the G and D bands of the amorphous carbon in the spectral ranges 1500-1600 cm^{-1} and 1350 cm^{-1} respectively. These bands usually dominate the Raman signal upon carbonization and appear as very broad peaks covering the whole range between 1200 and 1600 cm^{-1} [1].

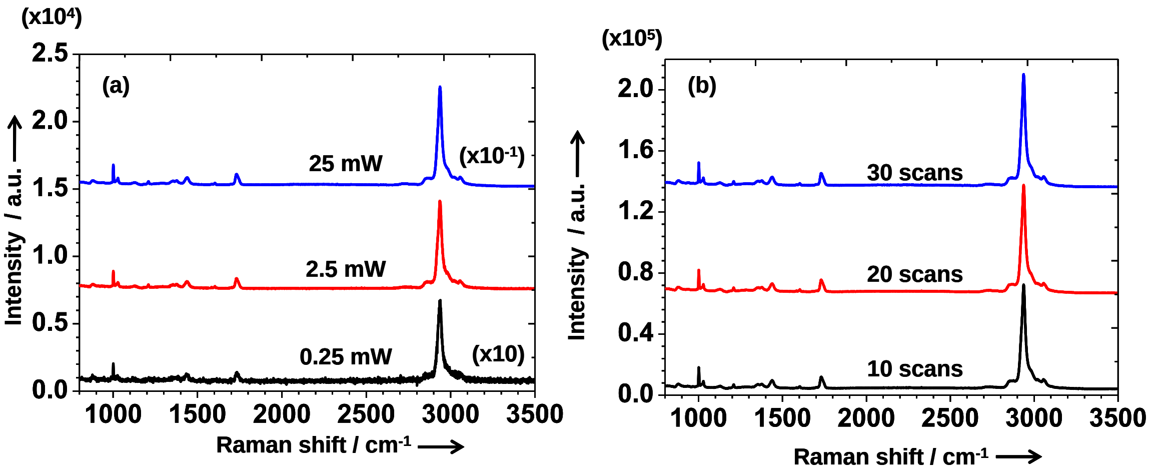

Figure S1. a) Raman signal from the same point on the sample for 0.25 mW, 2.5 mW, and 25 mW corresponding to 1%, 10%, and 100% of the maximum laser power. The excitation wavelength in all the measurements was 514 nm and the spot size for the maximum intensity of the laser is around 80 µm. The exposure time for each scan was 10 seconds. **b)** Raman signal of 2 µm PVAc layer on Ge substrate for different number of scans. All the measurements were recorded from the same position on the sample and the maximum laser power of 25 mW was applied.

For the Raman measurements, the cavities were prepared with bottom mirror of 30 nm thickness and the top mirror was sputtered on an area of about 200 µm in order to excite and collect locally in a small area of the cavity. The thickness of the top Ag mirror was in the range 10-15 nm in order to couple the excitation into the cavity and to decouple the Stokes scattered photons. The reference for Raman measurements was collected from an area without the top mirror on the same sample, thus both the reference and the cavity have essentially the same PVAc thickness. In order to characterize the IR absorption of the same area that was excited by Raman, there is a need to characterize locally the thickness of the cavity. For this purpose, all the sites measured with Raman were measured again in the visible region as detailed in the next section.

2. Samples characterization in the visible region

In order to locally characterize the cavity modes, the transmission in the visible region was acquired using microscope coupled to a cooled CCD camera. The transmission was measured at the same location where the Raman scattering was detected. Using the transmission in the visible, the real local thickness of the cavities and the mirrors were precisely determined. The physical thickness was then used to calculate the first mode of the cavity in the IR using the standard transfer matrices method for modeling the cavities. The absorption of the net polymer in the

visible was examined to confirm that there are no specific resonant bands in the measurements spectral range. Therefore, the background refractive index (RI) of the polymer was only considered for modeling the cavities and the references in the visible region.

3. Assessment of the Raman enhancement factors for the vibro-polariton states

In this section, we develop a simple approach that enables us to compare Raman polarizabilities of strongly coupled molecules with respect to the polarizabilities of non-coupled molecules. The difficulty stems from the fact that we only measure intensities which, in the case of strong coupling, are inevitable convoluted by the optical response (e.g. Fabry-Perot modes) of the cavity.

In order to deconvolute this response from the measured signals in the strong coupling situation, we have developed a simplified model where the Stokes photon is seen as being scattered from a vibrating dipole with an excited state given by the virtual state reached by the pump and a ground state determined by the energy level of the coherent vibro-polariton state. In this picture, this effective dipole was located at a specific position inside the cavity and emits photon with Stokes frequency after being excited. The mode structure at the excitation wavelength was used in order to determine the intensity at the location of the dipole. Given the optical excitation distribution function $\Gamma_{exc}(z_0)$ at position z_0 inside the cavity, the intensity of the scattered Stokes light $(I_S(z_0))_i$ from vibration i located at the same position, after averaging over all the orientations of the molecule will scale as follows [2] :

$$\text{(S1)} \qquad (I_S(z_0))_i \propto \Gamma_{exc}(z_0)\cdot(\omega_{exc}-\omega_i)^4\cdot\left\{\sum_{\rho,\sigma}\left|(\alpha_{\rho\sigma})_i\right|^2\right\}$$

where, as mentioned in the main text, $(\alpha_{\rho\sigma})_i$ is the $\rho\sigma$ matrix element of the polarizability tensor for transition i and ω_i is the frequency of the same vibrational mode. Although the actual intensity will be the result of averaging over the entire 4π solid angle, here we are interested in the scattered signal from the cavity compared to that from the reference. This radiation, generated by dipole which oscillates at frequency $\omega_S = (\omega_{exc}-\omega_i)$ induces a field at the dipole location $(E_S(z_0))_i \propto \sqrt{(I_S(z_0))_i}$ which then propagates through the optical structure. In the propagation

process, we calculated the multiple reflections of the scattered field at the boundaries of the structure assuming a plane wave pattern (See Fig. S2). The transmitted intensity, acquired on the detector, will linearly scale with the original scattered intensity and the transmission function of the structure.

Considering the propagation of the scattered field $(E_S(z_0))_i$ from the dipole as illustrated in Fig. S2, the ratio of the total intensity collected outside the structure to the scattered intensity from vibrational mode i reads:

$$(\gamma_{coll})_i \equiv \left|\frac{E_{tot}}{E_S(z_0)_i}\right|^2 = \left|\frac{t_1\left(1+r_2e^{2ik_zz_0}\right)}{1-r_1r_2e^{i2k_zL}}\right|^2 \quad ; \quad k_z = \frac{\omega_S}{c}n_c\cos\theta_c \qquad \text{(S2)}$$

where r_1 and t_1 are the reflection and transmission coefficients of the left boundary that involve the top small mirror in the case of the cavity or the PVAc\air interface in the case of the reference. The parameters r_2 , k_z, n_c, and θ_C are the reflection coefficient of the bottom mirror, propagation constant parallel to the cavity axis z, PVAc background RI, and propagation angle of the scattered photon inside the cavity respectively. Redefining the collection scheme effect on the scattered photon as $(\Gamma_S)_i \equiv (\omega_{exc}-\omega_i)^4\cdot(\gamma_{coll})_i$ and substituting in Eq. S1, one can express the total recorded intensity from vibration i as:

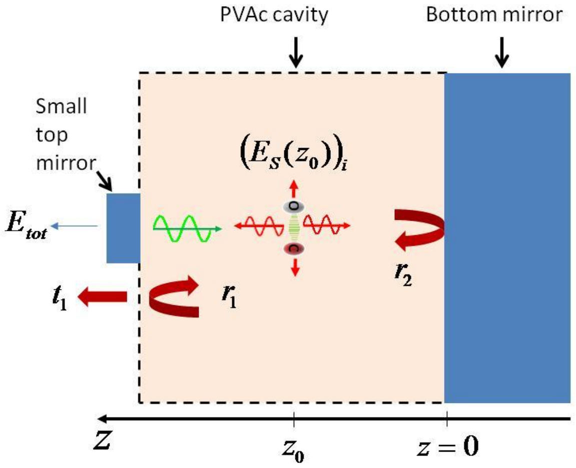

Figure S2. Schematic description of radiating dipole inside the cavity demonstrating the effect of the structure on the Stokes photon intensity when collected outside the structure. The reflection and transmission coefficients r_1 and t_1 respectively vary wither the signal is measured for the cavity or the reference.

(S3) $$\left(I_S\right)_i \propto \Gamma_{ex}\left\{\sum_{\rho,\sigma}\left|\left(\alpha_{\rho\sigma}\right)_i\right|^2\right\}\left(\Gamma_S\right)_i$$

Using Eq. S3, the polarizability amplification factors as defied in Eq. 2 in the main text can be estimated for the hybrid states VP+ and VP-. These values were deduced for one of the cavities used in the experiments to be $G_{\alpha VP+}=116$ and $G_{\alpha VP-}=1066$ for VP+ and VP- respectively. The later values correspond to the peak-to-peak value comparison. If the amplification factors are estimated by comparing the area under the peaks, these values can even reach $G_{\alpha VP+}=360$ and $G_{\alpha VP-}=5680$.

4. Composition of vibro-polariton states

For a fixed coupling strength g and a given detuning δ between the vibrational transition and the cavity mode, the vibro-polariton states simply write as $\left|VP+\right\rangle=\cos\theta\cdot\left|e\right\rangle\left|0\right\rangle+\sin\theta\cdot\left|g\right\rangle\left|1\right\rangle$ and $\left|VP-\right\rangle=-\sin\theta\cdot\left|e\right\rangle\left|0\right\rangle+\cos\theta\cdot\left|g\right\rangle\left|1\right\rangle$ where $\left|g\right\rangle$,$\left|e\right\rangle$ are respectively the ground (n=0) and first excited (n=1) vibrational states, and $\left|0\right\rangle$,$\left|1\right\rangle$ corresponding to the optical population of the cavity mode [3].

The relative weight of the components of each vibro-polariton states is given by $\tan 2\theta=-2\dfrac{g}{\delta}$.

This implies that as the detuning is increased by increasing the wave vector (in our case by increasing the numerical aperture), so does the contribution of $\left|g\right\rangle\left|1\right\rangle$ in the upper vibro-polariton $\left|VP+\right\rangle$. As a consequence, the Stokes signal is progressively depleted, due to the weakening of the weight of the vibration in $\left|VP+\right\rangle$. The opposite is true for $\left|VP-\right\rangle$ implying in this case that the strength of the Stokes signal is expected to be higher at larger angles than the Stokes signal associated with $\left|VP+\right\rangle$

Below, we associate $\left|e\right\rangle\left|0\right\rangle$ to a "vibrational content" while $\left|g\right\rangle\left|1\right\rangle$ is associated with a "photonic content". At resonance, the vibrational and photonic content is equal in both VP+ and VP-, namely each state will have 50% content of each the photon and the vibration. Changing the

resonance conditions create different proportions in the hybrid states. Fig. S3 show one example on PVAc cavity dispersion. Fig. S3a shows the dispersion of the uncoupled vibration and cavity modes (dashed lines) which are modified due to strong coupling to give the solid curves that describe the dispersion of both VP+ and VP-. The filled circles and diamonds give the measured energies for VP+ and VP- respectively. On Fig. S3b and c, one can see the photonic and vibrational contents of both VP+ and VP- respectively. Considering VP-, the photonic and vibrational weights at normal incidence are 0.42 and 0.58 respectively. By increasing the incidence angle, the photonic content of VP- decreases while the vibrational weight increases and approach unity toward high angles. The values at normal incidence are not equal because the cavity mode at 1770 cm^{-1} is slightly detuned with respect to the C=O vibrationl band at 1740 cm^{-1}. This explains the different intensity cross section of VP- and VP+ as discussed in the main text.

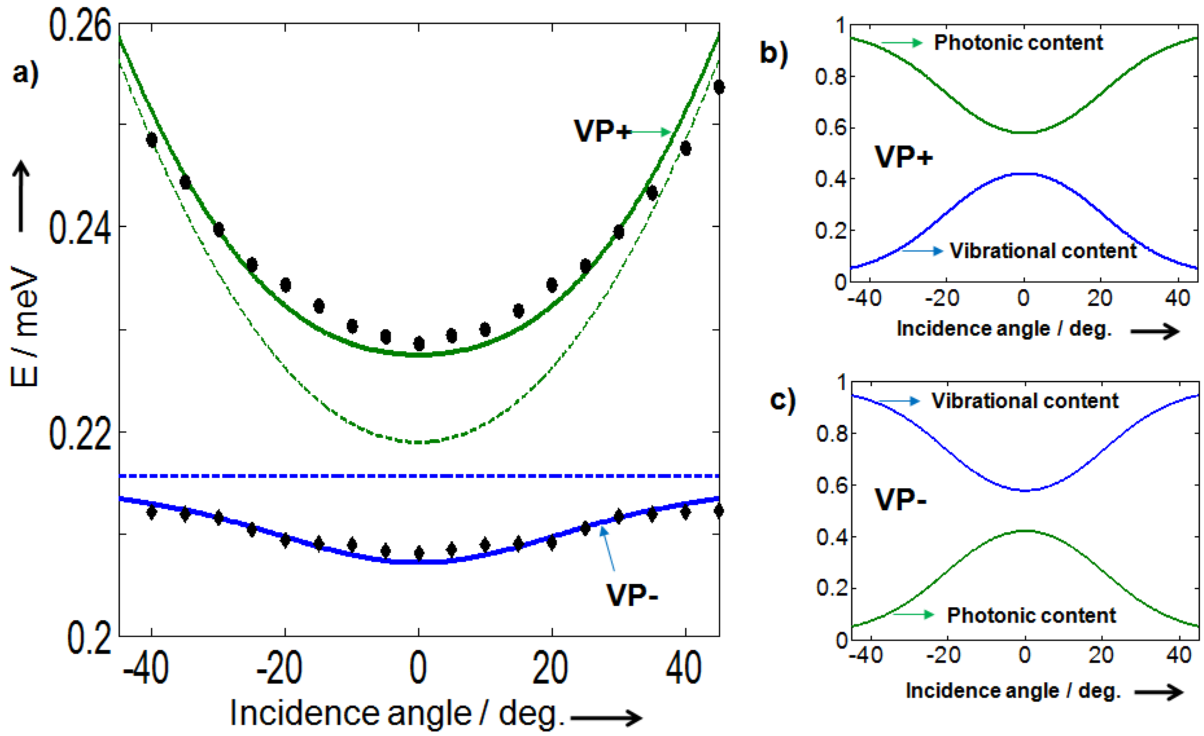

Figure S3. a) dispersion of PVAc cavity with uncoupled mode at 1770 cm-1 in normal incidence (dashed green curve) and uncoupled vibrational C=O mode at 1740 cm^{-1} (dashed blue line). The dispersion of the generated hybrid states VP+ and VP- are given by the green and blue solid curves respectively. The filled circles and diamonds are the measured dispersion of VP+ and VP- respectively. **b)** photonic (green) and vibrational (blue) weights in the hybrid state VP+. **c)** photonic (green) and vibrational (blue) weights in the hybrid state VP+.

5. References

[1] A. C. Ferrari, J. Robertson, *Phys. Rev. B.* **2000**, 61, 14095-14105.

[2] A. C. Albrecht, *Journal of Chemical Physics* **1961**, 34, 1476-1484.

[3] R. Houdré, *Phys. Stat. Sol. (b)* **2005**, 242, 2167-2196.
